# Comprehensive analysis of phage genomes from diverse environments reveals their diversity, potential applications, and interactions with hosts and other phages

**DOI:** 10.3389/fmicb.2025.1686402

**Published:** 2025-11-19

**Authors:** Chao Wei, Zhe Chen

**Affiliations:** National Key Laboratory of Pig Genetic Improvement and Germplasm Innovation, Jiangxi Agricultural University, Nanchang, China

**Keywords:** phage–phage interactions, phage diversity, potential applications, diverse CRISPR-Cas systems, diverse environments

## Abstract

Phages are ubiquitous and diverse, playing a key role in maintaining microbial ecosystem balance. However, their diversity, potential applications, and their interactions with hosts and other phages remain largely unexplored. To address this, we collected 59,652,008 putative viral genomes from our laboratory, 45 public viral datasets, and an integrated public viral genome database (IGN), covering seven habitats. We obtained 741,692 phage genomes with completeness ≥50% (PGD50), and most (93.83%, 695,938/741,692) of these phage genomes were classified into the Caudoviricetes class. We found that 158,522 species-level viral clusters that contained 28.96% (214,814/741,692) phage genomes without any known phage genomes in the IGN, indicating substantial novelty. Global phylogenetic trees for five iterations based on complete phage genomes significantly expanded the known diversity of the virosphere. Genome analysis revealed phage potential divergence with habitat types and highlighted the utilization of alternative genetic codes. Furthermore, 3D structural similarity searches demonstrated significant potential for annotating previously uncharacterized viral proteins. Analysis of CRISPR spacer inferred potential hosts of phages and competitive networks among phages, highlighting virulent phages as promising candidates for phage therapy against pathogenic bacteria. Intriguingly, diverse CRISPR-Cas systems were detected within phage genomes themselves, suggesting their enormous potential as novel gene editing tools. Collectively, this study provides a comprehensive phage genome resource, foundational for future research into phage–host and phage–phage interactions, phage therapy development, and the mining of next-generation genetic tools.

## 1 Introduction

Phages, ubiquitous, highly diverse viral components, are key regulators of microbial ecosystem balance, primarily through infection and lysis of bacteria and archaea (Clokie et al., 2011). They shape microbial community dynamics, metabolism, and diversity via established interactions (e.g., “kill-the-winner,” “piggyback-the-winner,” and evolutionary arms races) (Brown et al., 2022; Yan and Yu, 2024). Specifically, phages maintain diversity by lysing dominant strains, enhance host adaptability through horizontal gene transfer, and drive microbial diversification via adaptive co-evolution (Dion et al., 2020; Mangalea and Duerkop, 2020). Their therapeutic promise is exemplified in combating multidrug-resistant pathogens through phage therapy (Federici et al., 2022). Furthermore, phages engage in complex co-evolutionary dynamics with their hosts and environments. For instance, under heavy metal stress like chromium contamination in soil, phage–host interactions can shift from a predatory relationship to a potentially mutualistic one, with an increase in lysogeny and phage-mediated horizontal gene transfer potentially aiding host adaptation (Touchon et al., 2017). Similarly, in freshwater lake systems subjected to multiple environmental stressors, the complexity and stability of virus-bacteria interaction networks can be significantly reduced, altering the composition of viral auxiliary metabolic genes and consequently impacting ecosystem functions like carbon cycling (Wang T. et al., 2025). These findings underscore the critical role of environmental factors in shaping phage–host interaction networks. Although a number of phage genome databases have been established, the data remain largely fragmented and exhibit significant habitat-specific biases (Resch et al., 2024; Wang et al., 2024). However, a significant research gap persists because two key resources are lacking: a unified, high-quality genome resource for phages from diverse habitats, and a comprehensive understanding of the global-scale architecture of phage–host interaction networks. This gap fundamentally limits systematic ecological and evolutionary insights (Bignaud et al., 2025; Wang B. et al., 2025).

To counter phage predation, prokaryotes have evolved the CRISPR-Cas (Clustered Regularly Interspaced Short Palindromic Repeats) system, an adaptive immune mechanism that provides sequence-specific defense against invading nucleic acids (DNA and RNA) through the stages of adaptation, expression, and interference (Burstein et al., 2017; Makarova et al., 2020). Diverse CRISPR-Cas systems have been identified within metagenome-assembled genomes (MAGs) across specific prokaryotic phyla (Shmakov et al., 2015; Yan et al., 2018), and this suggests a rich landscape of interacting phage genomes. Phages themselves have been found to harbor CRISPR-Cas systems, inspiring novel gene-editing tools, a comprehensive overview of CRISPR-Cas systems across entire prokaryotic host and phage populations is still lacking (Pausch et al., 2020; Al-Shayeb et al., 2022). This gap hinders our understanding of the tripartite interactions among phages, other phages, and host bacteria or archaea, and their collective role in maintaining microbial community homeostasis.

Herein, to bridge these knowledge gaps, we present the construction and comprehensive characterization of the PGD50 database, a curated collection of high-quality phage genomes integrated from diverse habitats. The primary objective of this study is to employ this unified resource to systematically evaluate global phage diversity, evolutionary patterns, and ecological interactions, with an emphasis on uncovering novel phages and elucidating their functional traits. To address these objectives, we designed a series of targeted analytical approaches: (1) Taxonomic classification and phylogenetic analysis were applied to delineate evolutionary relationships and quantify phylogenetic novelty. (2) CRISPR spacer matching was leveraged to infer phage–host interaction networks and uncover competitive dynamics among phages. (3) Structure-based functional annotation enabled the prediction of protein functions beyond sequence homology, expanding the functional landscape of phage genomes. (4) Comparative genomics of CRISPR-Cas systems identified their diversity and potential activity within phage genomes. Together, these integrated approaches provide a multidimensional perspective on phage ecology and evolution, while also facilitating the identification of phage-encoded systems with potential biotechnological utility.

## 2 Methods

### 2.1 Construction of a phage genome database (PGD50)

To obtain phage genomes from diverse environments, we collected and combined putative viral contigs from our laboratory, 45 public viral datasets across 7 habitats, and an Integrated Genomic Database [IGN: IMG/VR (Camargo et al., 2023a), GenBank (Benson et al., 2013), NT (Sayers et al., 2022); Supplementary Tables S1, S2], yielding 59,652,008 contigs for analysis. Among them, we performed a dereplication step on all viral genomes included in the IGN database using MMseqs2 with the “easy-linclust -c 1.0 --min-seq-id 1.0” options, clustering them at 100% sequence identity to ensure non-redundancy. Viral contig identification utilized a custom pipeline developed by Nayfach et al. (2021b) based on four signatures: presence of viral protein families, absence of microbial protein families, viral nucleotide signatures, and multiple adjacent genes on the same strand. Briefly, to identify the presence of viral protein families, we constructed hidden Markov models (HMMs) for 23,841 viral protein families from the IMG/VR database, after excluding 1,440 families that are commonly found in microbial genomes or plasmids. Conversely, to confirm the absence of prevalent microbial protein families, HMM profiles were constructed for 16,260 families from the Pfam-A database, following the removal of 452 families that are also common in viruses. All protein sequences were searched against these HMMs using hmmsearch [HMMER v3.3.2 (Potter et al., 2018); parameters: -Z 1, *E*-value <1 × 10^−10^], with the database of the top hit determining the classification. Concurrently, viral nucleotide signatures were identified using VirFinder v.1.1 (Ren et al., 2017), which employs k-mer frequencies and machine learning. Genomic organization was assessed by calculating the strand switch rate (number of strand switches divided by gene count) for contigs with multiple adjacent genes. Finally, 9,607,235 viral contigs with genome size ≥3 kb were obtained for subsequent analysis.

Phage identification employed two complementary methods (Devoto et al., 2019; Al-Shayeb et al., 2020; Shang et al., 2022). First, protein sequences derived from contigs were annotated against Pfam-A (Mistry et al., 2021), TIGRFAM (Haft et al., 2003), and VOGDB^1^ databases using HMMER v3.3.2 (Potter et al., 2018) with the “hmmsearch -E 1e-5” parameter. Genomes required two or more genes containing virus-specific keywords (“capsid, phage, terminase, base plate, baseplate, prohead, virion, virus, viral, tape measure, tapemeasure neck, tail, head, bacteriophage, prophage, portal, DNA packaging, T4, p22, and holin”), exclusion of prokaryote-specific terms (“ribosomal protein, preprotein translocase, and DNA gyrase subunit A”), and at least one spacer match from bacterial or archaeal genomes. Second, we used PhaMer v1.0 (Shang et al., 2022; Hu et al., 2024) with default parameters, which applies a Transformer model for metagenomic phage prediction.

Removing false positives involved assessing bacterial universal single-copy orthologs (BUSCOs) ratios (Simao et al., 2015) and curated viral protein family modules (VPFs) ratios (Gregory et al., 2020). Genomes were retained only if they exhibited a BUSCO ratio <0.067, or a BUSCO ratio >0.067 with at least three VPF hits. Subsequent processing detected provirus boundaries, removed host bacterial sequence contamination, and evaluated genome completeness using CheckV v0.8.1 (Nayfach et al., 2021a). The final PGD50 database comprised 741,692 phage genomes with ≥50% completeness.

### 2.2 Lifestyle prediction and taxonomy assignment of phage genomes (PGD50)

We predicted phage lifestyles using BACPHLIP v0.9.3 (Hockenberry and Wilke, 2021), which classifies genomes as virulent (score <0.5), uncertain (score 0.5–0.9), or temperate (score >0.9). Since temperate phages exhibit both lytic and lysogenic states, we integrated prophages identified by CheckV with BACPHLIP-predicted temperate phages to define the final temperate category. Taxonomic assignment was performed using geNomad v1.7.4 (Camargo et al., 2023b), which leverages viral taxon markers covering most ICTV-recognized lineages.

### 2.3 Clustering phage genomes to species-level viral clusters and identification of potential novel phage genome clusters

We clustered 741,692 PGD50 genomes into species-level viral clusters using a greedy centroid-based algorithm (Roux et al., 2019; Nayfach et al., 2021b; Tomofuji et al., 2022; Zeng et al., 2024; Wei et al., 2025) with threshold criteria of 95% average nucleotide identity (ANI) and ≥85% genome coverage, as recommended by Roux et al. (2019). Clusters lacking any phage genomes from the IGN database were subsequently classified as novel phage genome clusters. Furthermore, we obtained all phage genomes from the PhageScope (Wang et al., 2024) database (total 873,718 genomes). To ensure a fair comparison with our PGD50 dataset (completeness ≥50%), we first processed the PhageScope genomes through CheckV, retaining only those with ≥50% completeness (446,062 genomes). These were then dereplicated at 100% average nucleotide identity using MMseqs2 (--min-seq-id 1.0 -c 1.0), resulting in a high-quality, non-redundant PhageScope reference set of 334,616 genomes. A comparative analysis at the species-level viral cluster was performed based on the PGD50 and PhageScope reference sets.

### 2.4 Performing global phylogenetic analysis for phage genomes based on five iterations

To evaluate the phylogenetic novelty and contribution of our obtained phage genomes within the global context of phage diversity, we conducted a large-scale phylogenetic analysis. This approach allowed us to quantify the phylogenetic distance (PD) between our genomes and established reference sequences, thereby assessing the expansion of the known evolutionary landscape.

Specifically, we combined 44,311 complete phage genomes from PGD50 with 5,658 reference complete phage genomes from the VMR database.^2^ The combined dataset was processed through a five-iteration phylogenetic workflow adapted (Low et al., 2019). First, duplicate genomes were removed using MMseqs2 v2.0 (“--min-seq-id 1.0 -c 1.0”) (Steinegger and Soding, 2017). Protein coding sequences were then predicted using Prodigal v2.50 (Hyatt et al., 2010). The resulting protein sequences were clustered with MMseqs2 (“--min-seq-id 0.3 -c 0.7”), yielding 353,315 protein clusters. Clusters containing ≥3 proteins were used to build HMM profiles with MUSCLE v3.8.1551^3^ and HMMER. These were supplemented with 77 existing Caudoviricetes HMM profiles from single-copy protein markers, generating a total of 219,604 phage-associated HMM profiles.

In each iteration, core HMM profiles were identified by scanning progressively refined genome subsets against all profiles using HMMER (*E*-value ≤1 × 10^−3^; coverage ≥50%). A profile was considered core if it was present in ≥10% of genomes, had an average copy number ≤1.2, and an average protein length >100 residues. For phylogenetic tree construction, gene markers in retained genomes were identified via HMMER searches (*E*-value ≤1 × 10^−3^) against the core HMM profiles (Nayfach et al., 2021b). Multiple sequence alignments of these markers were trimmed with trimAl v1.4.rev22 (Capella-Gutierrez et al., 2009), retaining fragments with <50% gaps. Genomes needed to possess ≥3 markers present in >5% of alignment columns to be included. The final phylogeny was reconstructed using IQ-TREE2 v2.1.3 (Nguyen et al., 2015) under the LG + F + G4 model with 1,000 ultrafast bootstraps, and visualized in iTOL.^4^ Finally, phylogenetic distances between genomes were computed from the resulting tree branch lengths using the ape v5.7-1 (Paradis and Schliep, 2019) and picante v1.8.2 (Kembel et al., 2010) packages in R, enabling quantitative assessment of the novel diversity introduced by our dataset.

### 2.5 Potential divergence analysis of complete phage genomes with habitat types

To minimize confounding effects from genomic fragmentation and unannotated habitats, we analyzed 26,439 complete phage genomes with verified habitat origins. These genomes were clustered into genus-level viral clusters based on average amino acid identity (AAI) and gene sharing (Nayfach et al., 2021b; Tomofuji et al., 2022; Zeng et al., 2024; Wei et al., 2025). Protein sequences were first predicted using Prodigal, followed by all-vs-all BLASTP alignments in DIAMOND v2.1.9.163 (Buchfink et al., 2015). For each phage pair, we calculated AAI percentages and shared gene proportions. Genome pairs exhibiting <50% AAI or <20% shared genes were clustered using MCL v14-137 (van Dongen, 2008) with an inflation factor of 2.0.

For clusters containing ≥4 genomes distributed across ≥2 habitats, we constructed phylogenetic trees (Wu et al., 2024). Core genes were identified using Roary v1.7.8 (Page et al., 2015) (-i 50 option) and aligned to create multi-FASTA files. Phylogenies were reconstructed with FastTree v2.1.10 (Price et al., 2010) and visualized in iTOL. Branch lengths between all genome pairs were systematically measured within each cluster. We performed two one-tailed Wilcoxon rank sum tests to compare branch lengths: (1) between genomes from identical habitats versus (2) between genomes from different habitats. Clusters where cross-habitat branch lengths significantly exceeded same-habitat distances (*p* < 0.05) were designated as exhibiting potential habitat-specific divergence.

### 2.6 Identifying alternative genetic codes in phage genomes (PGD50)

We employed a custom Prodigal v2.50 to identify open reading frames in all 741,692 PGD50 genomes using four genetic coding schemes: the standard genetic code (11) and three alternative codes—TAG recoding (15), TAA recoding (90), and TGA recoding (91) (Ivanova et al., 2014; Nayfach et al., 2021b; Lou et al., 2024). Briefly, for a phage with a genome size <100 kb, if its protein-coding density with the genetic codes 15, 90, or 91 increased >10% compared to that with the standard genetic code 11, we considered that this phage genome tended to use the corresponding alternative genetic code. For those phages with a genome size ≥100 kb, the threshold for considering the utilization of an alternative genetic code was the increase of protein-coding density >5%.

### 2.7 Functional annotation of phage genomes (PGD50)

We predicted proteins from all 741,692 PGD50 genomes using their corresponding alternative genetic codes and clustered them into 4,372,210 protein clusters via MMseqs2 (“--min-seq-id 3.0 -c 0.7”). To account for the mixed genetic repertoire of phages, which often includes genes of bacterial origin acquired via horizontal gene transfer, we utilized a multi-database approach including Pfam-A (Mistry et al., 2021), TIGRFAM (Haft et al., 2003), and VOGDB (see text footnote 1) for functional annotation to ensure broad coverage of both viral and bacterial protein domains. Representative sequences from each cluster were then functionally annotated against the three databases using HMMER (hmmsearch) (Potter et al., 2018) with an *E*-value threshold of 1 × 10^−5^.

To address unannotated proteins, we employed an approach of structural similarity searches developed by Nomburg et al. (2024) leveraging conserved structural domains from horizontal gene transfer events between viruses and cells. Given the substantial computational demands of structure prediction, our structural analysis was limited to representative sequences from the top 100 largest no-hit clusters. These structures were generated using ColabFold (Tunyasuvunakool et al., 2021), which leverages the AlphaFold2 algorithm. To infer functional insights, we performed structural alignments of our predicted models against the AlphaFold database using Foldseek (v1.0) (van Kempen et al., 2024). A TM-score threshold of ≥ 0.4 was employed to filter the alignments, retaining only those pairs with a statistically significant topological similarity for functional inference.

### 2.8 Host prediction for phage genomes (PGD50) and identification of phage–phage interactions

We predicted hosts for 741,692 PGD50 genomes through CRISPR spacer matching. CRISPR spacers were identified from microbial genomes and MAGs in GTDB (Genome Taxonomy Database) (Chaumeil et al., 2022), UHGG (Unified Human Gastrointestinal Genome) (Almeida et al., 2021), and pig gut (Chen et al., 2021) databases using MinCED v0.4.2^5^ with default parameters. Taxonomic classification of MAGs employed GTDB-tk v2.0.0 (classify_wf mode) (Chaumeil et al., 2022). Spacer-phage mapping used BLAST v2.12.0 + (BLASTn, -max_target_seqs 10,000,000 -dust no -word_size 8 -evalue 10) (Camacho et al., 2009), with matches requiring ≤1 mismatch and 100% alignment. Successful mappings indicated host-phage relationships. For pathogenic targeting analysis, we downloaded complete *Escherichia coli* and *Klebsiella pneumoniae* genomes from GenBank,^6^ removed duplicates using dRep v3.2.2 (-pa 0.9 -sa 1) (Olm et al., 2017), and annotated virulence factors (VFDB, http://www.mgc.ac.cn/VFs/), antibiotic resistance genes (CARD, https://card.mcmaster.ca/), and pathogenic bacterial proteins (PHI database, http://www.phi-base.org/).

We identified CRISPR spacers within PGD50 genomes using MinCED with default parameters and performed reciprocal BLASTn searches against all phage CRISPR spacers. Interactions were confirmed when spacers mapped to other phage genomes with ≤1 mismatch and 100% alignment. CRISPR-Cas systems in both phages and hosts were predicted using CRISPRCasFinder v 4.3.2 (Couvin et al., 2018) with default parameters. Furthermore, Cas12 proteins were obtained and the Cas12 phylogeny was reconstructed using IQ-TREE2 v2.1.3 under the LG + F + G4 model with 1,000 ultrafast bootstraps, and visualized in iTOL (see text footnote 4).

### 2.9 Statistical analysis

All statistical analyses were performed using R packages (v4.2.1).

## 3 Results

### 3.1 Characterization of phage genomes with completeness ≥50% (PGD50) from diverse environments

**Figure 1:**
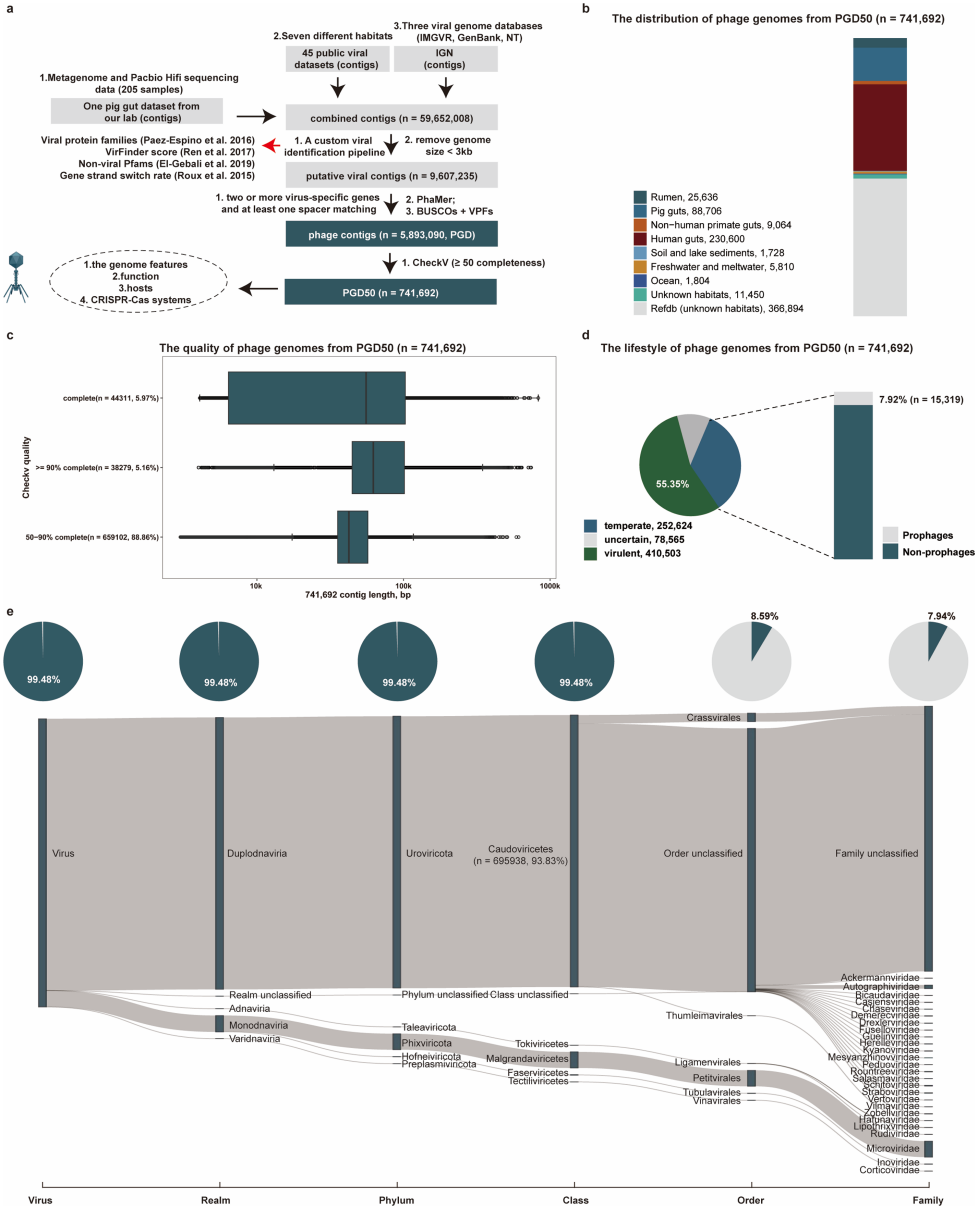
Identification and characterization of phage genomes from diverse environments. **(a)** The pipeline for identification of phage genomes (PGD50) from diverse environments. **(b)** The distribution of phage genomes (PGD50) in different habitats. Different colors represent phage genomes from different habitats. **(c)** The detailed distribution of genome length and quality for phage genomes (PGD50). **(d)** The lifestyle prediction of phage genomes (PGD50) and the proportion of prophages in temperate phages. The pie chart (left) represents different lifestyles of phage genomes with different colors, and the bar chart (right) shows the proportion of prophages in temperate phages. **(e)** The detailed taxonomy of phage genomes and proportion of known taxonomy for phages genomes at each taxonomic level. The Sankey diagram represents the detailed taxonomy of phages genomes and these pie charts show proportion of known taxonomy for phages genomes at each taxonomic level.

To expand phage genome recovery across habitats, we collected putative viral genomes from our laboratory, 45 public viral datasets, and an integrated public viral genome database (IGN). Using a custom pipeline, we identified 5,893,090 phage contigs from 59,652,008 total contigs based on: (1) using a custom viral pipeline, (2) removal of contigs with a genome size <3 kb, (3) retention of contigs encoding ≥2 virus-specific hallmark genes, (4) retention of contigs with ≥1 CRISPR spacer match, (5) using the PhaMer tool, and (6) confirmation using BUSCO and VPFs. Following validation using multiple methods, we retained 741,692 high-confidence genomes with completeness ≥50% (termed PGD50; Figure 1a).

We estimated the source distribution of phage genomes from PGD50, and found that 230,600 and 88,706 phage genomes were recovered from the human gut and pig gut, respectively (Figure 1b). Completeness assessment of phage genomes in the PGD50 identified 44,311 complete genomes, which represented a valuable resource for the known virosphere diversity (Figure 1c). We further focused on the lifestyle of phages in the PGD50 and found 55.35% (410,503/741,692) phage genomes were predicted as virulent phages, highlighting therapeutic potential against pathogenic infections (Figure 1d). Taxonomic analysis of phages in the PGD50 using geNomad assigned 93.83% (695,938/741,692) to the Caudoviricetes class, yet only 7.94% (58,902/741,692) achieved family-level resolution, demonstrating both substantial novelty and persistent classification challenges (Figure 1e and Supplementary Table S3).

### 3.2 Assessing novelty of PGD50 and global phylogenetic analysis of complete phage genomes

**Figure 2:**
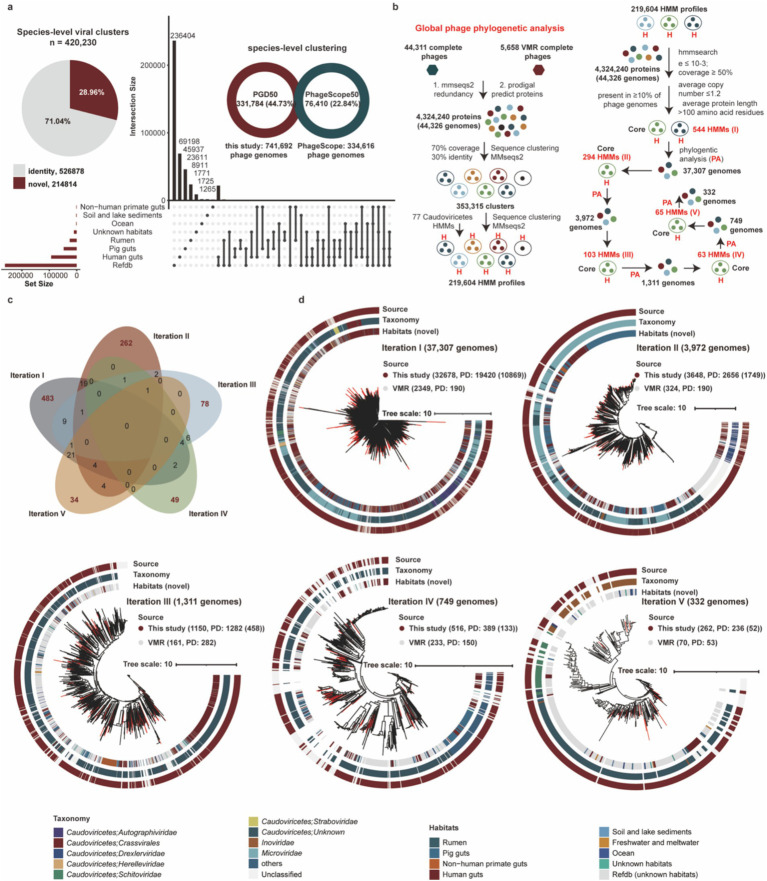
Assessing novelty of PGD50 and construction of global phylogenetic trees based on complete phage genomes. **(a)** The assessing novelty of PGD50 at species-level viral clusters. The pie represents the proportion of novel phages from PGD50 and the UpSet plot compares phage populations among different habitats. **(b)** The pipeline of global phage phylogenetic analysis of complete phages and core HMM profiles and number of phage genomes for five iterations. The pipeline (left) is performed to build all 219,604 HMM profiles based on the MMseqs2 and HMMER software. The pipeline (right) is performed to generate core HMM profiles and phylogenetic trees for five iterations, and the core HMM profiles for each iteration are generated based on all 219,604 HMM profiles. The parameters for generating core HMM profiles are shown in first iteration, and the parameters for five iterations are consistent. Phylogenetic trees for five iterations are constructed based on the method developed by Low et al. (2019) and corresponding core HMM profiles. **(c)** The sharing and unique core HMM profiles for five iterations to global phage phylogenetic trees. The distribution of sharing and unique core HMM profiles are described by the Venn diagram. The red numbers represent the unique core HMM profiles and the black number represent the sharing core HMM profiles for five iterations. **(d)** The global phage phylogenetic trees for five iterations and the source distribution of phage genomes from this study and the VMR database. The different colors OD outer circle for the phylogenetic trees represent phage genomes from different sources and the red clades represent novel complete phage genomes from this study.

To evaluate the novelty of phage genomes in the PGD50, we clustered 741,692 phage genomes into 420,230 species-level viral clusters at the threshold of 95% average nucleotide identity (ANI) and 85% coverage. We found 69,198, 45,937, and 23,611 species-level viral clusters were specifically identified in the human gut, pig gut, and rumen, respectively. Analysis of the species-level viral clusters confirmed the substantial novelty of our dataset. Specifically, 37.72% of the clusters themselves were novel, as they contained no sequences from the IGN database including IMG/VR, GenBank, and NT. These novel clusters comprised 28.96% of all the phage genomes analyzed (Figure 2a). Furthermore, a comparative analysis at the species-level viral cluster revealed the distinct contribution of our resource: 331,784 (44.73%) of the 741,692 genomes in our PGD50 dataset are not present in the PhageScope database. In contrast, only 76,410 (22.84%) of the 334,616 PhageScope genomes with completeness ≥50% are absent from our dataset. It demonstrated that our study has contributed a massive number of novel phage genomes that were absent from a leading, recently published database.

To resolve global evolutionary relationships of phages, we constructed phylogenetic trees for five iterations using 44,311 complete phages from this study and 5,658 reference genomes from the Virus Metadata Resource (VMR) from the International Committee on Taxonomy of Viruses (VMR_MSL40.v1). Briefly, we first obtained 353,315 protein clusters and 219,604 HMM profiles based on protein clustering and HMM profile generating using MMseqs2 and HMMER. Notably, we filtered and generated core HMM profiles for these complete phage genomes and performed five iterations to construct global phylogenetic trees (Figure 2b). Briefly, this iterative process was essential due to the vast diversity of our dataset. In each iteration, phage genomes not placed in the phage phylogenetic tree were identified, their specific marker genes were inferred, and these new markers were added to a composite set. This strategy progressively captured a broader spectrum of phage diversity, enabling a more inclusive and robust global phylogeny than would be possible with a single, static marker set. Interestingly, core HMM profiles of five iterations showed low inter-iteration similarity (Figure 2c), confirming representation of distinct phage diversity subsets. Phylogenetic distance (PD) metrics from all iterations (Figure 2d) collectively demonstrate significant expansion of known virosphere diversity.

### 3.3 Potential divergence analysis with habitat types using complete phage genomes

**Figure 3:**
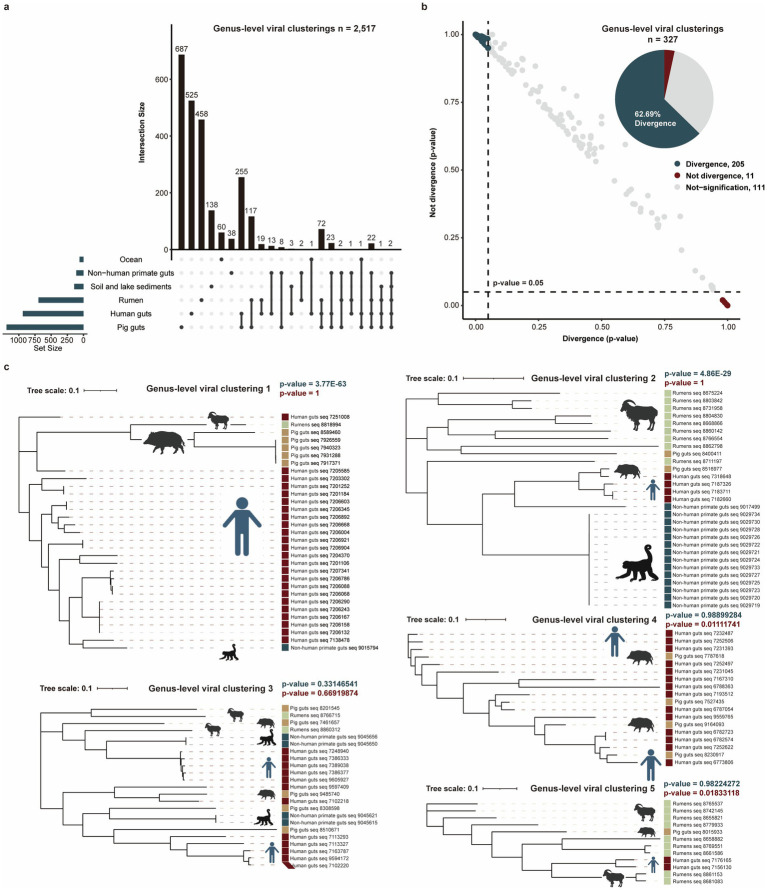
Potential divergence analysis for complete phage genomes with habitat types. **(a)** Comparing genus-level viral clusters among six habitats. **(b)** Potential divergence analysis of genus-level viral clusters with different habitat types. The dot plot shows the likelihood (*p*-values) distributions of 327 genus-level viral clusters that were diverged (black green dots) or not diverged (red dots) with habitat types. The pie chart shows the proportion of divergent and not-divergent genus-level viral clusters. **(c)** Five examples of genus-level viral clusters potentially diverged or did not diverge with habitat types. The blue *p*-value (*p* < 0.05) and the red *p*-value (*p* < 0.05) represent the significant divergence and significant not divergence for phage genomes with habitats.

To minimize impacts of the genomic fragmentation and unknown habitat, we analyzed 26,439 complete phage genomes from seven known habitats. These were clustered into genus-level viral clusters at the threshold of <50% average amino acid identity (AAI) or <20% of shared genes and an inflation factor of 2.0, yielding 2,517 genus-level viral clusters. Our analysis revealed a substantial number of habitat-specific genus-level viral clusters, with 687 uniquely identified in the pig gut, 525 in the human gut, 458 in the rumen, 138 in soil and lake sediments, 60 in the ocean, and 38 in the non-human primate gut (Figure 3a). These clusters contained no complete phage genomes from any other habitat, highlighting the distinct viral populations endemic to each environment.

To investigate potential divergence with habitat types, we analyzed 327 genus-level clusters containing ≥4 genomes distributed across ≥2 habitats (Supplementary Table S4). For each genus-level viral cluster, phylogenetic trees were constructed to test whether genomes from the same habitat exhibited closer evolutionary distances than cross-habitat counterparts. We observed that 62.69% (205/327) of clusters showed significantly closer phylogenetic distances among same-habitat genomes (*p* < 0.05), supporting potential habitat-phage divergence (Figure 3b). As the examples, genus-level viral clusters 1 and 2 demonstrated clear habitat-based phylogenetic clustering. No divergent pattern was detected in genus-level viral clusters 3–5, indicating taxon-specific variation in evolutionary dynamics (Figure 3c).

### 3.4 Functional potentials of phage genomes in the PGD50

**Figure 4:**
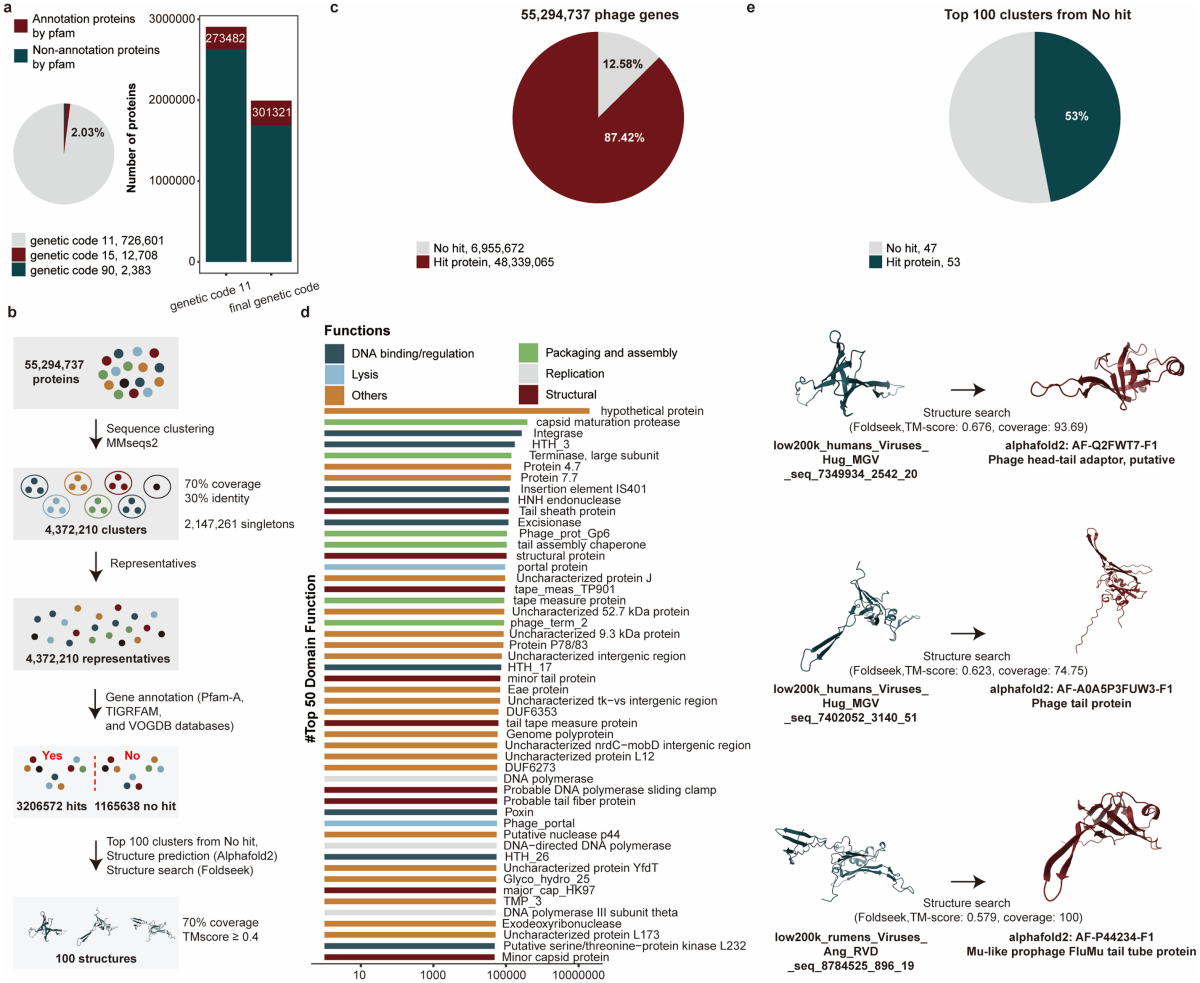
Functional annotations of phage genomes in the PGD50. **(a)** The proportion of using alternative genetic codes for phages genomes (pie chart), the number of proteins annotated by the Pfam-A database for phages using five alternative genetic codes (bar chart). **(b)** The pipeline of functional annotations for 55,294,737 phage genes. **(c)** The proportion of annotated phage genes for 55,294,737 phage genes. **(d)** Functional items with the numbers of phage genes in the top 50. Different colors represent different functional categories. **(e)** The proportion of further annotated phage genes using structure searching method for genes of top 100 clusters from no hit. The pie chart represents the proportion of annotated phage genes using structure searching method, and the 3D structures of phage genes were compared with Alphafold2 and Foldseek.

We investigated whether phages utilize alternative genetic codes to maintain low coding density and prevent protein fragmentation. Using custom Prodigal (v2.50), we evaluated four genetic codes (11, 15, 90, 91) based on total potential coding scores. The standard genetic code (11) dominated (97.97%, 726,601/741,692), while a small proportion (2.03%, 15,091/741,692) recoded stop codons as glutamine (Q, genetic codes 15) and Glycine (G, genetic codes 90). Notably, no genomes recoded TAA as glutamine (Q, genetic code 91) (Figure 4a). To identify phages employing alternative genetic codes, we applied a specific threshold during prediction. The use of the correct, corresponding genetic code for these identified phages then led to a significant improvement in functional annotation, as evidenced by a higher match rate against the Pfam-A database.

Viral proteins were highly divergent even within the same virus family, limiting the utility of sequence-based similarity searches when amino acid identity fell below 30%. To overcome the limitations of sequence-based annotation (e.g., for hits with <30% AA identity), we performed structural similarity searches. This approach leverages the fact that protein structural domains were often more conserved than their amino acid sequences, allowing for the detection of distant evolutionary relationships that were otherwise missed (Figure 4b). Interestingly, 87.42% (48,339,065/55,294,737) proteins were annotated (Figure 4c) and we further classified these genes into functional items, and the items with the number of annotated genes in the top 50 were listed. Among them, functional items related to the structure, assembly and packaging, DNA replication and transcription, and lysis, all of which were typical functional capacities of phages were enriched by annotated genes (Figure 4d). Critically, structural searches resolved 53% (53/100) of previously unannotated proteins (top 100 clusters with no sequence hits), demonstrating its power for annotating divergent viral proteins (Figure 4e and Supplementary Table S5).

### 3.5 Revealing phage–host relationships and pathogen targeting potential via CRISPR spacer matching

**Figure 5:**
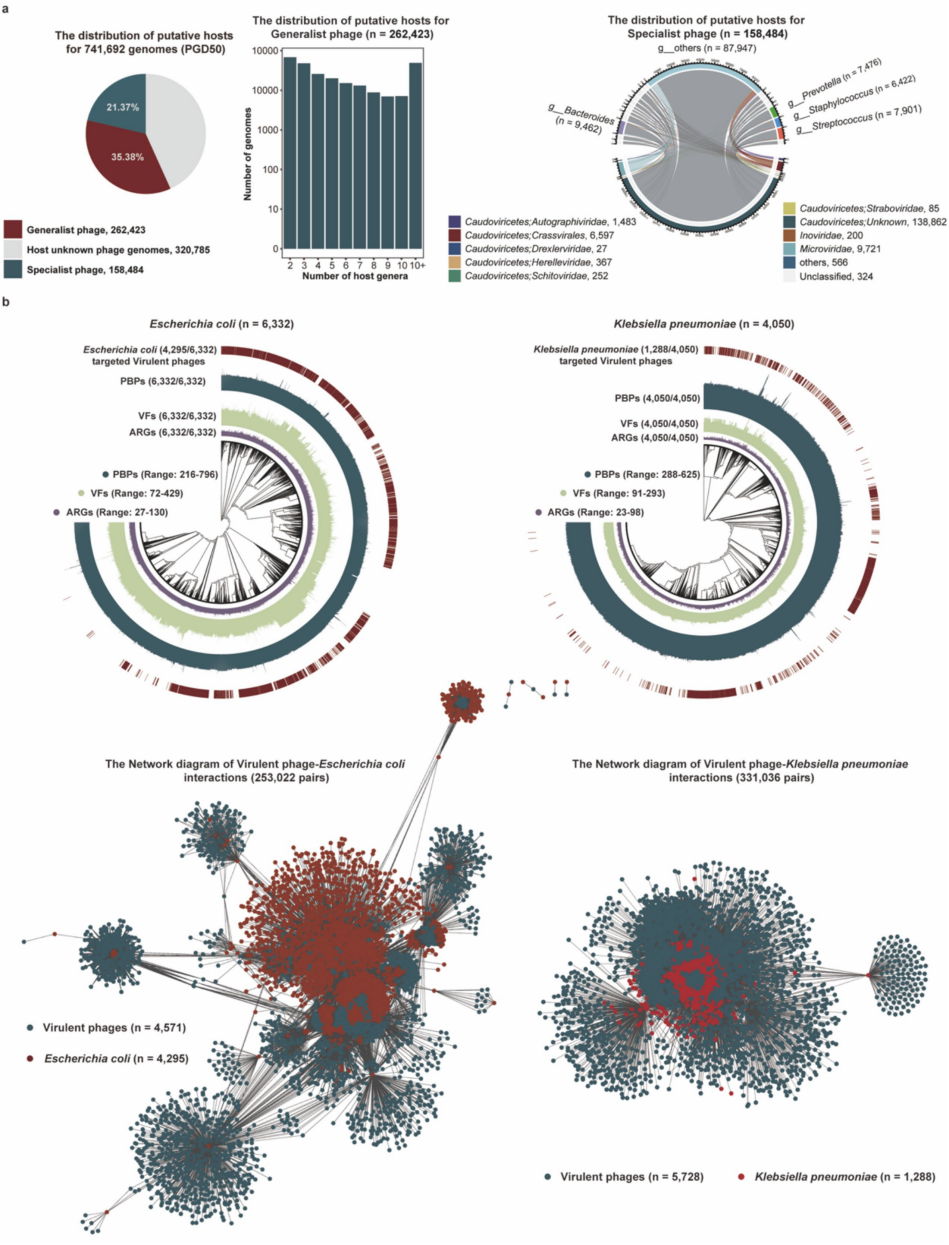
Potential hosts and targeting pathogen for phage genomes in the PGD50. **(a)** The proportion of generalist and specialist phages in the PGD50 (pie), the distribution of putative host numbers for 262,423 generalist phages at the genus level (box plot), and the distribution of putative prokaryotic hosts for 158,484 specialist phages (cycle diagram). **(b)** The phylogenetic trees of pathogenetic bacteria including *Escherichia coli* and *Klebsiella pneumoniae* and the network diagram of virulent phages targeting *Escherichia coli* and *Klebsiella pneumoniae*. The outer circle in phylogenetic trees represents *Escherichia coli* and *Klebsiella pneumoniae* genomes targeted by virulent phages, and the inner three circles represent *Escherichia coli* and *Klebsiella pneumoniae* genomes with VFs, ARGs, and PBP proteins, respectively.

The distribution of host bacteria or archaea is a strong determinant for the distribution of phages, and the indigenous phage community also greatly affects the structure and function of the host bacterial or archaeal community. To establish phage–host linkages, we leveraged CRISPR spacer similarity, a key determinant linking phage distribution to their bacterial or archaeal hosts. Analysis of 741,692 phage genomes identified putative hosts for 56.75% (420,907/741,692) phages through spacer matches (Supplementary Table S5). Our analysis revealed that 35.38% (262,423/741,692) of phage genomes were linked via CRISPR spacers to multiple bacterial genera, with some connections spanning different phyla (Figure 5a). There were 21.37% (158,484/741,692) phage genomes only targeting one host genus, and host for these specialist host viruses mainly belonged to the keystone genera *Bacteroides* and *Prevotella*, critical in gut or hypersaline ecosystems.

Our analysis focused on *Escherichia coli* and *Klebsiella pneumoniae* given their predominant role in the global burden of antimicrobial resistance. This focused approach allows for a deeper investigation into phage solutions for these clinically paramount threats (Murray et al., 2022). We analyzed virulent phages targeting pathogens including *Escherichia coli* and *Klebsiella pneumoniae*, and first estimated the distribution of PBP proteins, VFs, and ARGs in collected pathogenic bacteria genomes from the GenBank database. Interestingly, we found virulent phage genomes in the PGD50 could target 67.83% (4,295/6,332) *Escherichia Coli* and 31.08% (1,288/4,050) *Klebsiella pneumoniae* based on CRISPR spacer matching (Figure 5b), suggesting that these virulent phages in the PGD50 might be an ideal tool for phage therapy via targeted lysis of pathogenic bacteria.

### 3.6 Competitive phage networks and CRISPR-Cas system distribution

**Figure 6:**
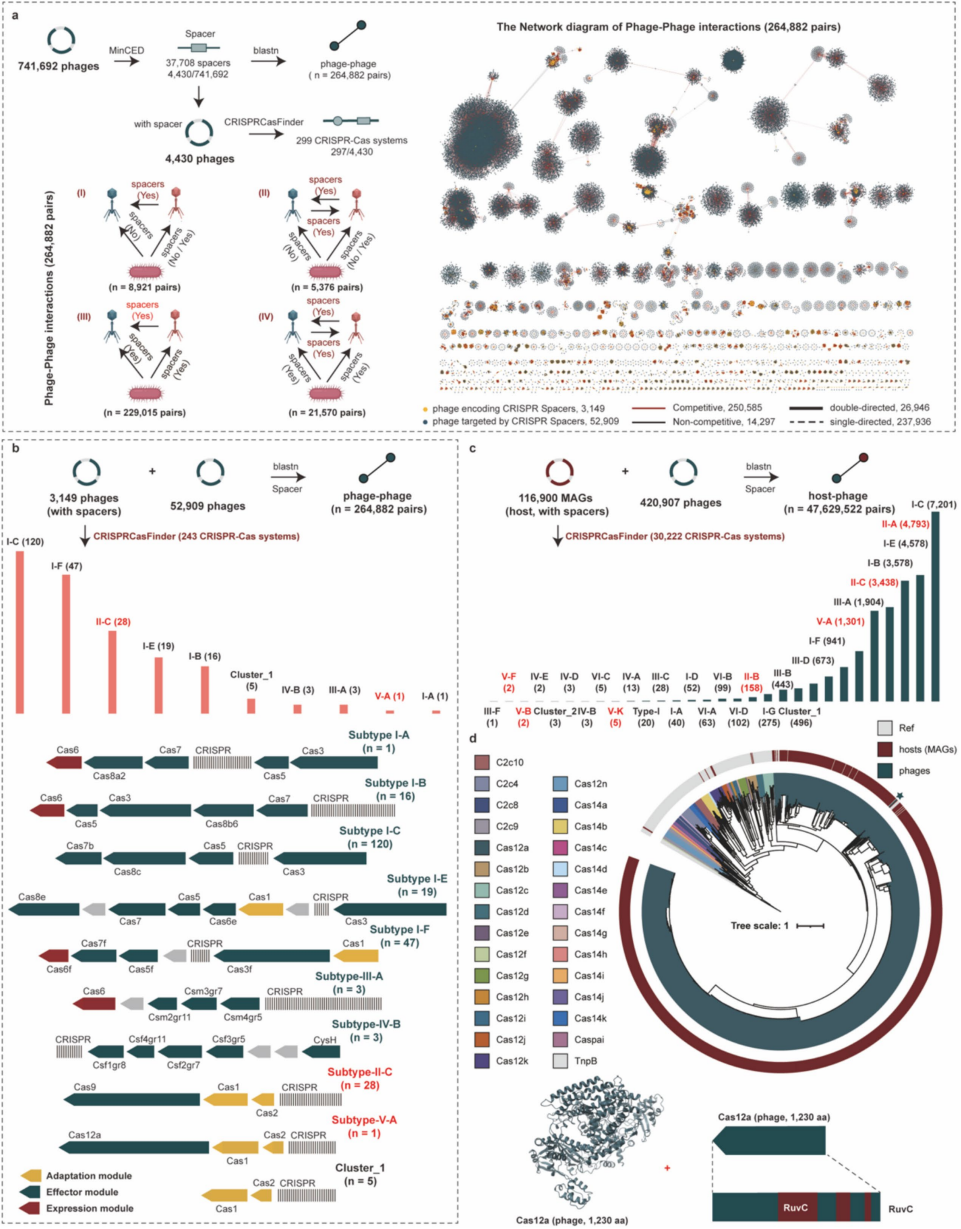
Phage–phage interaction and revealing diverse CRISPR-Cas systems for hosts and phages. **(a)** The pipeline of identification for CRISPR-Cas systems and CRISPR spacers for phage genomes, and the four specific phage–phage interactions (left). The network diagram of phage–phage interactions for phage genomes (right). **(b)** The detailed distribution and structural diagram of CRISPR-Cas systems for phage genomes. **(c)** Distribution of CRISPR-Cas system types across host genomes involved in predicted phage-host interactions. **(d)** The phylogenetic tree of Cas12 proteins from phages, hosts, and reference proteins, and the 3D structure and RUVC domain of the Cas12a protein from phage genomes. The different colors of outer circle in the phylogenetic tree represent the sources of Cas12 proteins, and the colors of clades represent different types of Cas12 proteins.

We identified 37,708 CRISPR spacers within 4,430 phage genomes in the PGD50. Among these, 8.35% (3,149/37,708) targeted 52,909 phage genomes, establishing extensive phage–phage interaction networks (Figure 6a). Target pair analysis revealed single-directed relationships (where one phage targets another without reciprocal targeting) dominated these interactions at 89.83% (237,936/264,882), while double-directed pairs (reciprocal targeting) constituted the remaining 10.17%. Critically, 94.60% (250,585/264,882) of targeted pairs consisted of phages infecting the same host, revealing a high prevalence of potential competitive relationships (Supplementary Table S6).

CRISPR-Cas systems are adaptive immune systems widespread in hosts but rarely found in phage genomes. We identified 243 CRISPR-Cas systems within phage genomes that specifically target other phages, with the most prevalent subtypes being I-C, I-F, and II-C (Figure 6b and Supplementary Table S7). Among these, 37 systems (15.23%) were complete. More broadly, a total of 299 CRISPR-Cas systems were identified across all phage genomes. In contrast, we found 30,222 CRISPR-Cas systems encoded by host genomes, which were predominantly subtypes I-C, II-A, and I-E (Figure 6c and Supplementary Table S8). Notably, phage-encoded CRISPR-Cas systems (83.61%, 250/299) frequently lacked spacer acquisition proteins (Cas1, Cas2, and Cas4), suggesting partial horizontal gene transfer (HGT) during acquisition. Furthermore, focusing on the two most prevalent subtypes, we found that 96.97% (116/120) of the I-C systems and 17.86% (5/28) of the II-C systems were missing these Cas proteins. Given the biotechnological significance of Cas12 proteins, particularly their compact size for gene-editing applications, we performed a phylogenetic analysis to explore their diversity in phages. Our analysis incorporated a total of 1,311 Cas12 sequences, comprising 1,310 derived from host genomes (spanning subtypes Cas12a, Cas12b, Cas12f, and Cas12k) and a single, notable Cas12a sequence identified from a phage genome in our study. The resulting phylogenetic tree (Figure 6d) revealed distinct clustering of Cas12 subtypes, with the phage-encoded Cas12a nesting within the diversity of host-encoded Cas12a sequences. This placement suggested a potential evolutionary history of horizontal gene transfer between phages and their bacterial hosts for this particular system. While the single phage sequence precluded a conclusion on phage-specific diversity, its presence alone was significant. The Cas12 protein identified in the phage contained the conserved RuvC nuclease domain. This domain was critically important as it was responsible for the DNA cleavage activity that formed the foundation for all DNA-targeting applications of Cas12 in biotechnology (Makarova et al., 2020). The preservation of this functional domain in the phage protein underscored its potential as a functional nuclease and a valuable resource for mining novel gene-editing tools.

## 4 Discussion

Phages play a key role in maintaining the balance of microbial ecosystems (Rascovan et al., 2016; Emerson et al., 2018), but their interactions with hosts and other phages are largely unknown. This study presents a comprehensive and expansive resource of phage genomes, significantly augmenting our understanding of global phage diversity, evolutionary dynamics, and functional potential. By integrating massive datasets from diverse habitats, we have assembled a collection of 741,692 medium-to-high-quality phage genomes, vastly exceeding the scale of most previous individual studies and significantly enriching existing public databases like the IMG/VR (Roux et al., 2021), GenBank (Benson et al., 2013), and NT (Sayers et al., 2022) (IGN). The sheer number of phage genomes analyzed here provides unprecedented resolution for exploring the virosphere.

The most striking finding is the immense proportion (28.96%) of phage genomes clustering into 158,522 species-level viral clusters that lack any representatives in the IGN. This underscores a profound gap in our current cataloging of viral diversity. These novel species-level viral clusters likely represent phages endemic to understudied environments, highly divergent lineages, or those infecting uncultured hosts. Their discovery dramatically reshapes our perception of the virosphere’s true breadth and complexity, suggesting that known phages represent merely a fraction of the total diversity. The construction of global phylogenetic trees reveals that our dataset substantially expands the known diversity of the *Caudoviricetes*, filling critical phylogenetic gaps and introducing novel, deep-branching lineages. This expansion was particularly pronounced in previously underexplored habitats such as the pig gut and rumen. However, we must acknowledge that this apparent “expansion” is partially shaped by the inherent unevenness of existing genomic databases. The deeper sequencing of certain environments like the human gut naturally allows for the resolution of finer-scale genetic diversity, while the true evolutionary breadth of under-sampled habitats likely remains underestimated (Nayfach et al., 2021b). Consequently, the present evolutionary map should be viewed as a robust yet interim framework, one that will be refined as future metagenomic surveys encompass a broader spectrum of global ecosystems.

Beyond cataloging diversity, our genomic analyses revealed distinct evolutionary patterns reflected in the association between phages and their habitats. While the observed signals are consistent with potential divergence with habitats, we interpret these patterns as strong evidence of environmental filtering and habitat adaptation. Phages are likely finely tuned to the physicochemical and biological conditions of their respective niches, a phenomenon driven by factors such as host availability, nutrient constraints, and inter-phage competition (Koskella and Brockhurst, 2014; Sharma et al., 2018). However, caution is warranted in ascribing these distribution patterns solely to strict co-evolution. Habitat filtering, where environmental conditions selectively favor both compatible hosts and their phages represents a powerful, alternative mechanism shaping these ecological relationships (Lennon and Martiny, 2008). In other words, the signal we detect may reflect phage adaptation to their host’s ecological niche, rather than direct, synchronous genome evolution between phage and host. For instance, a longitudinal study of *Aeromonas* and its phages demonstrated that their interaction dynamics oscillated over time between “arms race” and “fluctuating selection” modes (Marston et al., 2012). Our cross-sectional study may have captured only a single snapshot of this complex, dynamic process. Disentangling these possibilities will require future longitudinal time-series sampling of the same habitats, combined with experimental validation through controlled co-evolution experiments of specific host-phage pairs (McGrath, 2024). Furthermore, the detection of alternative genetic code usage in some phages highlights an intriguing evolutionary strategy (Devoto et al., 2019), possibly conferring advantages like evasion of host defenses or optimization of replication efficiency under specific conditions, warranting deeper investigation.

The functional annotation of phage genomes, particularly for proteins with no known homologs, remains a major challenge (Nomburg et al., 2024). Our application of 3D structural similarity searches represents a significant methodological advance. By moving beyond sequence-based homology, this approach provided functional predictions for 53% of the top 100 previously unannotated viral proteins based on 3D structural resolution. This not only enhances our understanding of the functional repertoire encoded within this novel phage diversity but also provides a powerful strategy for future viral metagenomic studies.

Our analysis of phage–host interactions, leveraging CRISPR spacer matching, provided crucial insights into the ecological networks connecting phages and their potential hosts (Tisza and Buck, 2021; Johansen et al., 2023). A key finding was that a substantial proportion (35.38%) of phage genomes were linked via spacer matches to hosts spanning multiple genera or even phyla, suggesting the potential for broad host ranges (Nishijima et al., 2022; Bignaud et al., 2025). However, these *in silico* predictions warrant a nuanced interpretation. True ecological generalists capable of productively infecting distantly related hosts are considered rare in nature. The observed patterns may therefore stem from several alternative factors: the presence of common integrative genetic elements shared across diverse hosts, the inherent limitations of predictive bioinformatics, or the fact that spacer matches can reflect past, non-productive infection events rather than active, concurrent replication. Despite these important caveats, this spacer-based approach proved highly valuable for generating specific, testable hypotheses, robustly predicting potential hosts for numerous phages, including those with links to clinically relevant pathogenic bacteria (López-Beltrán et al., 2024) and thereby highlighting promising candidates for further therapeutic exploration. This approach not only revealed complex ecological networks of phage competition and co-existence mediated through shared CRISPR targets (Tisza and Buck, 2021) but also proved particularly valuable for identifying strictly lytic (virulent) phages with therapeutic potential. The strictly lytic life cycle of these virulent phages makes them ideal therapeutic candidates, as it enables the direct and rapid eradication of target pathogens. However, translating these foundational discoveries into clinical practice faces significant challenges. Two of the most prominent hurdles are the typically narrow host range of phages, which can limit their applicability against diverse bacterial strains, and concerns regarding the potential transduction of bacterial virulence factors (Petrovic Fabijan et al., 2023). The vast and diverse reservoir of virulent phages uncovered in our study provides a unique resource to address these challenges. The many genes of unknown function within these genomes may encode novel proteins capable of modulating or evading host immune responses. Through rational genetic engineering, such as modifying phage tail fibers to broaden host range or knocking out highly immunogenic, non-essential genes, we can leverage these natural blueprints to develop next-generation phage-based therapeutics that are safer, more effective, and better suited to clinical application (Meile et al., 2022).

A particularly exciting and unexpected finding was the detection of diverse CRISPR-Cas systems within the phage genomes themselves (Al-Shayeb et al., 2020; Al-Shayeb et al., 2022). Phage-encoded CRISPR-Cas systems open fascinating new avenues for research into phage–host arms races, where phages may utilize these systems to compete against other mobile genetic elements (including other phages) or even manipulate host defenses (Al-Shayeb et al., 2020). Beyond their biological significance, these phage-borne systems represent a vast, largely untapped reservoir of novel CRISPR-Cas variants with potentially unique properties (e.g., smaller size, different PAM specificities) (Pausch et al., 2020; Carabias et al., 2021; Al-Shayeb et al., 2022). This positions our phage genome collection as an extraordinarily rich source for mining the next generation of gene editing tools with enhanced capabilities for biotechnology and medicine.

While this study provides a landmark resource for viral ecology, several limitations inherent to metagenomic analysis must be acknowledged. First, our reliance on a genome completeness threshold (PGD50) ensured high-quality analysis but may have systematically excluded abundant, fragmented viral sequences, leading to an underestimation of the diversity of certain phage groups. Second, although our functional inference was augmented by structural similarity searches to reveal distant homologies (van Kempen et al., 2024), it remains constrained by homology-based methods; proteins with truly novel folds represent a fundamental blind spot, and all predictions require biochemical confirmation. Finally, our host prediction strategy relies solely on CRISPR spacer matches. While this method provides high-specificity links, it is inherently limited by the incompleteness of microbial genome catalogs and reflects historical infection events rather than active replication. Furthermore, this singular approach leaves phage interactions with many uncultured or un-sequenced hosts undetected; future work incorporating complementary methods, such as k-mer composition analysis, would be essential to systematically expand host assignment coverage and obtain a more comprehensive view of phage–host interaction networks. Future efforts combining more permissive assembly strategies, multi-faceted host prediction, and experimental validation will be crucial to overcome these biases. In total, this study delivers an unparalleled genomic resource that fundamentally expands our knowledge of phage diversity on Earth. We have uncovered a vast reservoir of novel phages, revealed intricate patterns of potential divergence and adaptation, developed innovative methods for functional annotation, and uncovered critical insights into phage–host interactions and competitive networks. Most significantly, we have demonstrated the immense, dual application potential of this resource: firstly, as a targeted library for discovering potent phage therapy agents against pathogenic bacteria, and secondly, as a treasure trove for mining the next generation of innovative CRISPR-Cas-based gene editing technologies. This dataset provides an essential foundation for future research aimed at understanding the intricate roles of phages in global ecosystems, combating antibiotic resistance, and advancing genetic engineering.

## 5 Conclusion

In conclusion, this study constructed the PGD50 database, a unified resource of 741,692 high-quality phage genomes, which enabled a systematic reassessment of global phage diversity and ecology. Our key advance lies not in the initial reporting of phage diversity or phage-encoded CRISPR-Cas systems, but in the substantial expansion of their documented scale and diversity. Specifically, we identified a significant number of novel, deep-branching lineages, represented by 158,522 species-level viral clusters that were absent from existing references. Furthermore, our analysis reframed the observed ecological patterns not as definitive “co-evolution,” but as a distinct “habitat divergence.” This pervasive phylogeographic signal indicates a strong environmental imprint on phage evolution, which may arise from co-evolutionary dynamics, environmental filtering, or a combination of both. Beyond diversity, our integrated approach including combining structural annotation, CRISPR spacer analysis, and comparative genomics, provided foundational insights into phage function, host interaction networks, and the expanded distribution of CRISPR-Cas subtypes within phages, underscoring their potential as future therapeutic and biotechnological tools. Collectively, this work provides a refined framework and resource for future research into phage biology and application.

## Data Availability

The datasets presented in this study can be found in online repositories. The names of the repository/repositories and accession number(s) can be found in the article/Supplementary material.

## Footnotes

^1^ http://vogdb.org

^2^ https://ictv.global/vmr

^3^ http://www.drive5.com/muscle/

^4^ https://itol.embl.de/

^5^ https://github.com/ctSkennerton/minced

^6^ https://www.ncbi.nlm.nih.gov/genbank/
