# Supplementary material for: Comprehensive analysis of phage genomes from diverse environments reveals their diversity, potential applications, and interactions with hosts and other phages
Source: Front Microbiol. 2025 Nov 19;16:1686402. doi: 10.3389/fmicb.2025.1686402 (PMC12676247; doi:10.3389/fmicb.2025.1686402)
Supplement: Supplementary file 1 [file Supplementary_file_1.docx]

**Supplementary Tables**

Table S1. The summarized numbers of viruses, phages identified in each study.

Table S2. The data sources used in this study for phage genomes from pig guts.

Table S3. The detailed information on completeness, lifestyle, taxonomy, and genetic codes for 741,692 phage genomes in the PGD50.

Table S4. The exact P values (n = 327) for potential divergence of phage genomes with habitat types.

Table S5. The detailed information of top 100 for no hit protein clusters using Foldseek.

Table S6. The distribution and competing types of phage-phage interactions in the PGD50.

Table S7. The distribution of CRISPR-Cas systems (n = 299) for phage gnomes in this study.

Table S8. The distribution of CRISPR-Cas systems (n = 30,222) for hosts targeted phages in this study.
